# Communicative Participation in Adolescents and Young Adults: A Concept Elicitation Study

**DOI:** 10.1111/1460-6984.70069

**Published:** 2025-06-30

**Authors:** Eline Alons, Nicole ter Wal, Margreet R. Luinge, Caroline B. Terwee, Ellen Gerrits, Lizet van Ewijk

**Affiliations:** ^1^ Research Centre Healthy and Sustainable Living HU University of Applied Sciences Utrecht Utrecht the Netherlands; ^2^ Department of Languages, Literature and Communication Institute for Language Sciences (ILS), Utrecht University Utrecht the Netherlands; ^3^ Research Centre Healthy Ageing; Youth, Education and Society Hanze University of Applied Sciences Groningen Groningen the Netherlands; ^4^ Epidemiology and Data Science Amsterdam UMC Locatie VUmc Amsterdam the Netherlands; ^5^ Amsterdam Public Health Research Institute, Methodology Amsterdam the Netherlands

**Keywords:** adolescents, communicative participation, item bank, patient reported outcome measure, young adults

## Abstract

**Background:**

Communicative participation is the primary outcome of speech and language therapy for people with communication disorders. However, there are no instruments that measure communicative participation from the perspective of adolescents and young adults. Moreover, little research has been conducted in which adolescents and young adults with various communication problems were asked about relevant participation situations in which they need to communicate. Before a new measurement instrument can be developed, it is necessary to explore adolescents’ and young adults’ views on their communicative participation.

**Aims:**

In this study we identified situations in which adolescents and young adults experience barriers participating because of communication problems, as well as situations in which they have no difficulties. In addition, we identified aspects of these situations that influence communicative participation in order to gain a deeper understanding of concepts related to communicative participation.

**Methods and Procedures:**

This concept elicitation study concerned in‐depth interviews on self‐experienced communicative participation, in which diaries were used as a sensitizing exercise. Participants were asked to keep a diary for 1–2 weeks in which they described their communicative participation during the day. They were asked to describe situations in which they were not able to participate because of their communication disorder, and to describe situations in which they were able to participate. Within 1 week after completing the diary, participants were invited for an in‐depth interview, in which the content of the diary was discussed.

**Outcomes and Results:**

Twelve participants were included in this study (7 females, 5 males; 13–27 years) with a variety of communication disorders, including language disorders, speech disorders, hearing loss, or a combination of them. They described a total of 234 situations related to different domains (e.g., communicative participation in school or with friends). Out of these situations, 37 concepts that influence communicative participation were found that were related to the six categories: person (e.g., strangers), topic (e.g., figurative language), pace (e.g., time pressure), location (e.g., school), moment (e.g., energy) and mode (e.g., group conversations).

**Conclusions and Implications:**

The results of the study provide understanding in adolescents’ and young adults’ perspectives on communicative participation. The identified situations form the basis for developing an item bank for measuring communicative participation in adolescents and young adults with communication disorders. In upcoming studies, the items will be extensively assessed on the quality aspects of comprehensiveness, comprehensibility and relevance.

**WHAT THIS PAPER ADDS:**

**What is already known on this subject**

- Communicative participation is the most important outcome of speech and language therapy. However, measurement instruments (preferably patient reported outcome measures, PROMs) to assess communicative participation of adolescents and young adults are scarce. Moreover, perspectives of adolescents and young adults on self‐experienced communicative participation, which can lead to the development of a measurement instrument, have not yet been identified.

**What this paper adds to existing knowledge**

- This study is about the self‐experienced communicative participation situations mentioned by adolescents and young adults themselves. It presents 37 concepts describing communicative participation that were identified from communicative participation situations mentioned by adolescents and young adults themselves.

**What are the potential or actual clinical implications of this work?**

- This study provides more insight into concepts that describe communicative participation from the perspectives of adolescents and young adults. The results will be used to develop an item bank measuring communicative participation in the target population.

## 1 Introduction

With the rise of person‐ and child‐centred care, increased focus has been placed on *communicative participation* as the main outcome of speech and language therapy (Baylor and Darling‐White 2020; Cunningham et al. 2016). Communicative participation is defined as ‘taking part in life situations in which knowledge, information, ideas, or feelings are exchanged’ (Eadie et al. 2006, 309). Despite the fact that this definition was published in 2006, treatment planning and evaluation of speech and language therapy still often focuses on the body functions and activity domains of the International Classification of Functioning, Disability and Health (ICF; WHO 2001; Cunningham et al. 2016; Torrence et al. 2016). The meaningful participation of individuals is often overlooked. One of the barriers to this is the lack of assessment tools designed to measure communicative participation (Kwok et al. 2023). For adults, Baylor and colleagues (Baylor et al. 2013) developed the Communicative Participation Item Bank (CPIB): a patient reported outcome measure (PROM) for communicative participation. A review by Darling‐White (2017) revealed that at that time there were no PROMs for measuring communicative participation in children and adolescents. In addition to this review, a systematic search was conducted searching for all PROMs and parent reports that tap into the construct of either communication or participation (Alons et al. 2024). In this search, nine PROMs were identified. However, none of these instruments specifically measured communicative participation. Upon reviewing the items of the identified PROMs against the criteria for communicative participation, it was found that although no PROM focused exclusively on this construct, 74 items did address aspects related to communicative participation. The identified PROMs were each developed for use with a specific population or communication disorder, rather than all focusing on the same condition. While these instruments serve important purposes within their intended contexts, our point is that none of them offer a unified approach to measuring communicative participation across diverse populations. To support child‐centred care in speech and language therapy, there is a need for a PROM that captures communicative participation from the perspective of children and adolescents and is applicable across a variety of communication disorders (Darling‐White 2017; Cunningham et al. 2016).

When developing a PROM, the most important measurement property is content validity (i.e., the degree to which the content of a measurement instrument is an adequate reflection of the construct to be measured; Mokkink et al. 2010). Items should be both relevant and comprehensive for the construct to be measured (De vet et al. 2011). It is therefore important that items are generated based on direct input from the target population for whom the PROM is intended (Patrick et al. 2011; Terwee et al. 2018) to ensure relevance of items and comprehensiveness of the total set of items. This information needs to be elicited from the target population itself, since the experiences of participation are deeply personal and subjective, and contain components that are not visible to others (e.g., parents or teachers), such as confidence, effort, and feelings included in interactions (O'Halloran and Larkins 2008).

Some research has been conducted on the impact of communication problems on adolescents’ and young adults’ daily lives. In their qualitative study on adolescents with developmental language disorder (DLD), Ekström et al. (2023) explored their experiences with language and communication in a school context. Participants reported on a range of educational, social and emotional challenges, which they perceived to be influenced by both individual characteristics and abilities, as well as situational, contextual and social factors. Cobb et al. (2019) interviewed adolescents who stutter on their school experiences and found that these adolescents do not always feel supported in their communication by the school environment and that this is related to the level of peer support, teacher support and general knowledge about stuttering. Connaghan et al. (2022) explored the communication and social interaction experiences of adolescents with congenital motor speech disorders due to cerebral palsy or Down syndrome. Besides the communication impairment itself, they found that environmental factors (e.g., physical environment and attitudes of the communication partner) influenced social communication. In deaf/hard of hearing adolescents, Zaidman‐Zait and Dotan (2017) found four main domains that adolescents mentioned as problematic when being asked about everyday problems, consisting of participation in school, interaction with parents, interaction with peers and leisure activities. In the work of Kelly et al. (2018), adolescents with autism spectrum disorder (ASD) were interviewed about their social communication skills. They found that conversations with peers in a school‐context and conversations with unfamiliar people were found to be very difficult, and that adolescents with ASD experience anxiety, rejection and isolation when in contact with peers. Although the terminology used in these studies varied (e.g., social participation, school participation, peer interactions), they are all linked to the construct of communicative participation and show that the impact of communication problems on a young person's daily life is significant. All of these studies show that successful communication is not only about the disorder, but also about personal and environmental factors that should be taken into account in both research and clinical practice.

Although these studies provide valuable insight into the impact of communication disorders on aspects of daily life, they all focus on one or a few aspects of daily life (e.g., peer relationships, school, everyday stressors) or a subpopulation of communication disorders (e.g., DLD, ASD, hearing, stuttering). For the purpose of developing a relevant and comprehensive PROM for measuring communicative participation, there is a lack of an overview of all key participation situations that require communication that are relevant for adolescents and young adults with various communication disorders.

Participation‐related constructs, including communicative participation, are typically person‐specific and context sensitive. The difference between adults and children may be relatively clear; adult participation (e.g., managing the household or working) is different from children's participation (playing with friends or going to school) (Darling‐White 2017). However, within children, participation is also likely to change (rapidly) as a child grows up. It is likely that a 9‐year‐old child will report different participation situations than a 16‐year‐old teenager. Where a 9‐year‐old might mention playing with peers, a 16‐year‐old might report on having a first job. The contexts in which a person participates depend, amongst other things, on an individual's life stage. These life stages are described in the Life Course Health Development (LCHD) framework (Halfon and Hochstein 2002), and consist of preconception, early childhood (birth to about 6 years), middle childhood (about 6–11 years), adolescence (about 12–18 years), and emerging adulthood (about 18 to mid/late 20s). Using life stages in research on participation is essential in order to be able to tailor care to the individual needs of people at different stages of life.

Prior research has explored the types of communication situations that are difficult for adults. Ter Wal et al. (2023) identified six themes, all with several underlying concepts, that influence communicative participation. In this study no distinction was made between adulthood and emerging adulthood. The youngest participant was 27 years old, followed by the second youngest at 39 years old. In addition, in the work of Baylor et al. (2013) on the CPIB, the studies that were used to develop the initial item pool were conducted in populations aged 38–80 years old (Baylor et al. 2005, 2007; Yorkston et al. 2007). This means that the perspectives of young adults are not necessarily captured in prior work on measurement of communicative participation. A further exploration of people in emerging adulthood is therefore important, to ensure their participation contexts and difficulties are explored and made explicit.

In summary, for the development of a PROM, we need to identify the perspectives of children, adolescents and young adults with communication disorders on their self‐experienced communicative participation. Taking into account the life stages of this population, this study focuses on the elicitation of the perspectives of adolescents and young adults with communication problems on their communicative participation, by using a similar approach as Ter Wal et al. (2023). The aim of the present study was twofold: first, to identify relevant, self‐experienced situations in which adolescents and young adults have difficulties participating because of communication problems, as well as situations in which adolescents and young adults have no difficulties. Second, we aimed to identify relevant aspects of these situations that influence communicative participation.

## 2 Method

This concept elicitation study comprises qualitative research consisting of semi‐structured interviews combined with a diary approach.

### 2.1 Participants

A purposive sampling strategy was used to include adolescents and young adults aged between 12 and 25 with speech disorders (such as stuttering or verbal dyspraxia), language disorders (such as DLD), voice disorders, or with hearing loss. We aimed to include at least 12 participants with at least two participants per type of communication disorder (speech, language, hearing or voice) and to continue recruitment until no new key concepts appeared (Patrick et al. 2011). Participants were recruited in three ways: (1) through emailing the personal network of the research team, consisting of SLTs and researchers that work with children and adolescents within this population; (2) through social media posts on LinkedIn, Facebook page for Dutch SLTs and the Instagram account of author EA; (3) through messages via professional associations (Dutch Association of Speech and Language Therapy) and patient associations.

The following sociodemographic variables were collected: age, gender, diagnosis (as mentioned by the participant) and relevant comorbidities. Twelve participants were included in this study (7 females, 5 males) with a variety of communication disorders, including language disorders, speech disorders, hearing loss, or a combination of them. There were no participants with voice disorders. The ages varied from 13 to 27 years old (nine young adults, three adolescents). The categorization of participants as either adolescents or young adults was based on the Dutch school system. Those attending secondary school were classified as adolescents, while participants who had finished secondary school were classified as young adults. The mean age of the participants was 18 years old. The characteristics of the participants are presented in Table 1. All participants participated verbally in the interview. In two interviews, participants preferred the presence of their mother during the interview.

**TABLE 1:** Participant's characteristics.

PP	Age	Life stage	Gender M/F	Diagnosis	(Relevant) Co‐morbidities	Chosen diary method	Location of interview	Provided communication support
1	21	Young adult	F	Verbal dyspraxia, stuttering, developmental language disorder, mainly expressive	None	Photos, videos	External location; University of applied sciences	None
2	22	Young adult	F	Developmental language disorder, mainly expressive	None	Notes in phone	External location; University of applied sciences	None
3	23	Young adult	M	Hearing loss, bilateral	None	Written diary using Word	Online; Microsoft Teams	None
4	27	Young adult	M	Stuttering	None	Written diary using Word	External location; University of applied sciences	Interview time extended
5	25	Young adult	F	Cleft lip and palate, hearing loss	None	Written diary using Word	Participant's home	None
6	26	Young adult	F	Developmental language disorder, receptive and expressive	PDD‐NOS, ADHD	Written diary using Word	Participant's home	Adapting language, written support
7^*^	18	Young adult	F	Developmental language disorder, receptive and expressive	None	Written diary	Participant's home	Adapting language, written support
8	24	Young adult	M	Developmental language disorder, receptive and expressive, stuttering	None	Written diary using Word	Participant's home	Adapting language
9	13	Adolescent	F	Developmental language disorder, receptive and expressive	None	Notes in phone	Participant's school	Adapting language, written support, drawings
10	14	Adolescent	M	Developmental language disorder, receptive and expressive	None	Notes in phone	Participant's school	Adapting language, written support, drawings
11	19	Young adult	F	Progressive hearing loss	Visual impairment, Dutch second language	Written diary	Participant's school	Voice amplifier
12^*^	13	Adolescent	M	Developmental language disorder, receptive and expressive	None	Written diary using Word	Participant's home	Adapting language, written support, drawings

Abbreviations: F = female, M = male.

^*^Participants’ mother was present during the interview.

### 2.2 Ethical Considerations

This study was approved by the Internal Ethical Review Board (Reference Number 211‐000‐2022 of the HU University of Applied Sciences Utrecht) and was conducted according to the principles of the Declaration of Helsinki (World Medical Association 2013) and the Dutch Medical Research Involving Human Subjects Act.

All participants were provided with a letter of information about the study. Three versions of letters for participants were generated: (1) A letter for children 12–16 years old, age appropriate and supported with images for visual information; (2) A letter for adolescents and young adults > 16 years with normal wording; (3) A letter for adolescents and young adults with language problems, with adjusted wording and visual support using images. In addition, an information video was available explaining the aim of the study and what was expected of the participant.

In the Netherlands, for participants under the age of 16, parental consent must be obtained next to the consent of the participant. Therefore, a separate information letter was written to inform parents.

All participants and (if applicable) their parents provided written informed consent before participating in the study.

### 2.3 Communicative Participation

For this study, we used the definition of Eadie et al. (2006, 309) for communicative participation: ‘taking part in life situations in which knowledge, information, ideas, or feelings are exchanged.’ Eadie describes that communicative participation includes ‘a communicative exchange between at least two communicative partners (i.e., a message with the opportunity for a response) in the context of a life situation’ (Eadie et al. 2006, 312). We further specified these criteria in earlier research (Alons et al. 2024). For the communicative exchange, we added that the communication partner needed to be a natural person that communicates either directly in the here and now or over a prolonged period and physical distance (e.g., in texting a friend, emailing a teacher). To be about the context of a life situation, the situation had to entail performing a social role within a social context. This could either be because of a specific communication partner or by mentioning a specific location.

### 2.4 Data Collection

This study involved in‐depth interviews about self‐experienced communicative participation using diaries as a sensitizing exercise. The data collection was carried out by the first author, EA (SLT and PhD student). EA received training and coaching in interviewing through a professional course and coaching from colleagues in the research team. As an SLT herself, EA was experienced in communicating with people with communication difficulties. She was able to support communication whenever necessary.

Contact between the EA and the participant began as soon as the participant expressed interest via email or an online registration form. The information letter was sent to the participant, and the participant was invited to a short introductory meeting, either in person or online. During this meeting, EA introduced herself and explained the purpose of the study. The participant was given the opportunity to ask questions and decide whether or not to participate. If the participant decided to participate, data collection was started.

#### 2.4.1 Diary Method

Participants were asked to keep a diary for 1–2 weeks in which they described their communicative participation during the day. They were asked to describe situations in which they were not able to participate because of their communication disorder, and to describe situations in which they were able to participate.

The diaries were used as sensitizing exercise. Their use enabled participants to think about and reflect upon their communicative participation prior to having a conversation about it. The participants had to send their diary prior to the interview, to inform the topic list of the interview.

Participants were allowed to choose their own preferred way of keeping the diary. For example, a written diary using a provided format, making photos or videos, keeping notes on their phones or recording voice clips. The choice of diary‐method was discussed during the introduction meeting.

#### 2.4.2 Semi‐Structured Interview

Participants were invited for an interview, preferably 1 week after completing the diary. The interview took place at a location preferred by the participant. Presence of significant others (mainly parents) was allowed, but it was highlighted that the purpose of the interview was to gain the participant's experience on the topic. If necessary, the researcher supported the conversation by using conversation techniques, for example, facilitating drawings, writing keywords during the conversation, and speaking in short sentences. All interviews were audio‐recorded. In addition, field notes were made of situations that were mentioned before and after audio recording.

The interview guide was informed by the concept elicitation guideline of Patrick et al. (2011) and is presented in Appendix 1. The interview guide was discussed with authors LE (senior researcher and SLT) and ML (senior researcher and neurolinguist).

At the beginning of the interview, the interviewer asked a few warm‐up questions to build rapport. These questions were about the experiences of keeping the diary. After that, the diary was discussed, and questions were asked to gain more understanding of the situations described and aspects that affect the situation. In addition to the diary, the interviewer asked about a typical day in the participant's life. Participants were asked to reflect on their communication during their daily life participation. Each interview ended with a question where participants were asked to compare their life with the lives of peers, and to identify whether there are situations in which peers participate that they do not because of their communication disorder. With this question, there was also an opportunity to bring up contexts in which the participant does not participate at all because of limitations in communicative participation.

### 2.5 Analysis

Analysis was based on the interviews and the diaries. The diaries initially informed the interviews. In some cases, the diaries contained more than just a (short) description of the situation; they also included a direct reflection on the situation. This reflection was not always repeated in the same level of detail during the interview, but instead, additional questions were asked. If this was the case, the entire reflection from the diary was included in the analysis.

All interviews were transcribed verbatim by EA. After the interview, discussed situations were summarized and sent to the participant for a member check. Participants were asked if the described situations were accurate and whether we addressed all situations that are relevant for them. To ensure participants would be able to understand the member checks, short sentences were used, and structure was provided, by clustering situations by category (e.g., all school examples together). Two participants did not respond to the member check. One participant responded that he thought of a few more situations after the interview and sent a description of these per email. These were also included in the analysis. All other participants agreed with the content of the member check.

Data analysis was performed by authors EA and NW (PhD student and SLT). The first two transcripts were coded by two authors independently and discussed. The other transcripts were coded by EA.

**FIGURE 1:**
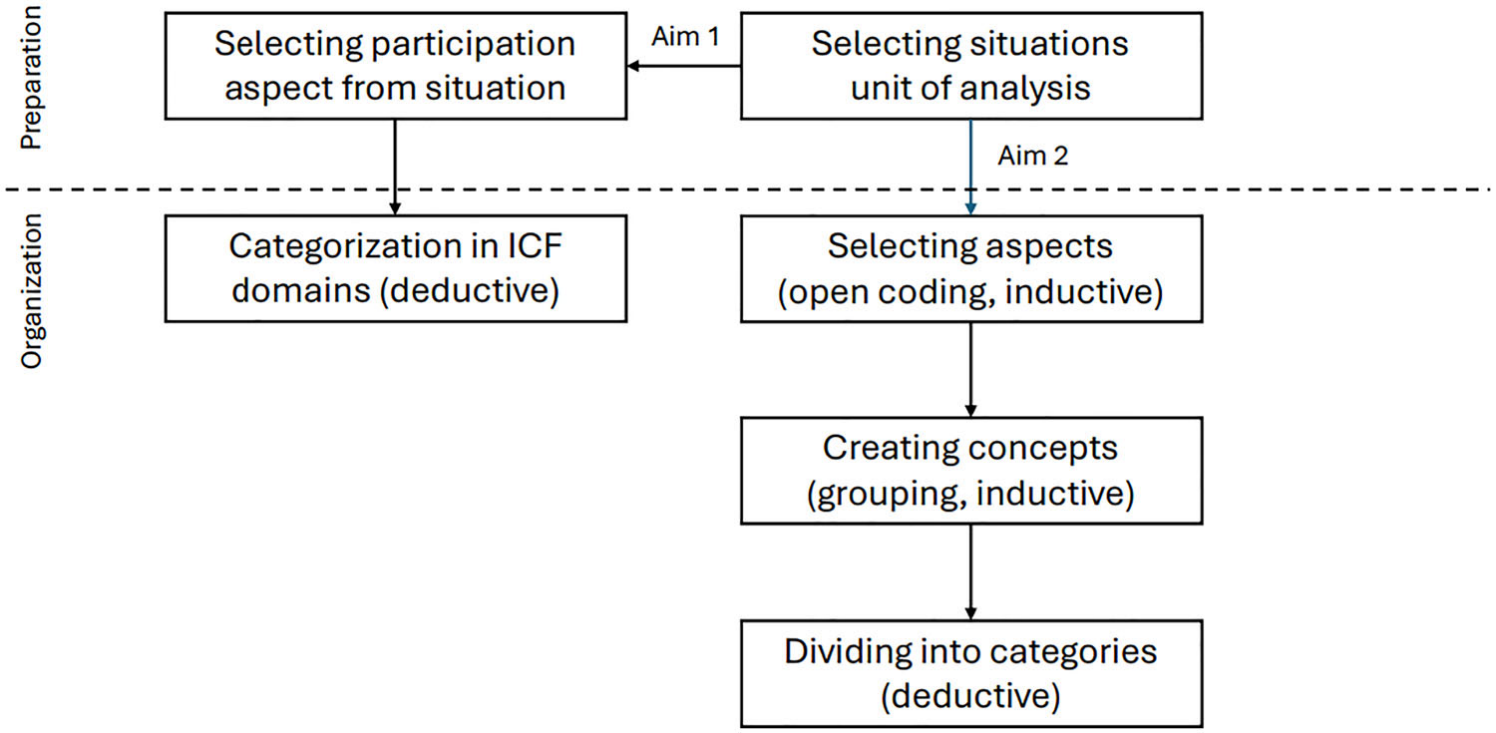
Steps in the analysis.

The data were analysed on manifest content using content analysis, which can be used to describe a phenomenon in a conceptual form (Elo and Kyngäs 2008) and allows words to be distilled into fewer content‐related categories. The analysis procedure was inspired by the work of Elo and Kyngäs (2008), although their suggested procedure was not strictly followed, as they also report that there are no simple guidelines for analysis, as each research project is unique (Elo and Kyngäs 2008). Figure 1 provides a detailed description of the steps taken during the analysis process.

The analysis started with selecting units of analysis, for which we used the situations that were mentioned by the participant (either based on the diary, or on the additional questions). Based on these units of analysis, analysis continued for both aims separately.

#### 2.5.1 First Aim: Communicative Participation Situations Described by the Participants

The first aim was to identify relevant, self‐experienced situations in which adolescents and young adults have difficulty participating because of communication problems, as well as situations in which they have no difficulties. The unit of analysis consisted of a situation that met the definition of communicative participation. In the next step, the situations were categorized by the situations into one of the domains of the ICF activities and participation chapter (organizing phase). This chapter contains nine domains: (1) learning and applying knowledge, (2) general tasks and demands, (3) communication, (4) mobility, (5) self‐care, (6) domestic life, (7) interpersonal interactions and relationships, (8) major life areas, and (9) community, social and civic life. EA and NW performed this step of categorizing the situations together. The situations were categorized for the sample combined, as well as for adolescents and young adults as separate groups.

#### 2.5.2 Second Aim: Relevant Concepts of Communicative Participation Situations

The second aim was to gain deeper understanding of communicative participation, by identifying relevant concepts that influence the mentioned situations. This analysis was chosen based on previous knowledge of the construct *communicative participation* from the research of Ter Wal et al. (2023). In their research, concepts that describe communicative participation were derived and formed the main outcome of the analysis. These concepts were then clustered into six themes, described in an empirical way (e.g., The person with whom one communicates largely determines the participant's ability to participate; the topic being discussed plays a role in being able to participate in a social situation). However, in the tables the authors describe, they use abbreviated categories, consisting of person, topic, pace, location, moment and mode (Ter Wal et al. 2023). These categories represent a framework that is, in our opinion, generic and thereby applicable in both adults and children. However, the context in which participation takes place will differ between adults and children, resulting in age‐specific concepts describing communicative participation. Based on this reasoning, we chose to code the units of analysis inductively to concepts and then used a deductive approach to code the concepts into the categories of Ter Wal et al. (2023). The steps of open coding and clustering the aspects into concepts were performed by EA and NW together. Hereafter, the concepts were categorized into one of the six categories of Ter Wal et al. (2023): person, topic, pace, location, moment and mode.

Elo and Kyngäs (2008) describe the categorization matrix as a tool to perform this last step. A categorization matrix was developed, representing the categories *person*, *topic*, *pace*, *location*, *moment* and *mode*. In case a concept would not fit within one of these categories, an inductive approach was carried out to create a new category.

## 3 Results

### 3.1 Communicative Participation Situations Described by the Participants

The participants reported a total of 234 situations in which they needed to communicate. Table 2 provides an overview of the identified domains and examples of codes that were classified within each domain. Most situations (95/234, 40.6%) were classified in ICF domain 8, major life areas. These situations concerned going to school or participating in work. 86/234 situations (36.8%) were classified in ICF domain 7, interpersonal interactions and relationships. Other situations were classified in domain 4, mobility (6/234, 2.6%); domain 5, self‐care (9/234, 3.8%); domain 6, domestic life (14/234, 6.0%); and domain 9, community, social and civic life (24/234, 10.3%). No situations were classified into domains 1, 2 and 3.

**TABLE 2:** Identified items divided in ICF domains activities and participation.

ICF domain activities and participation	*N* situations all participants (%)	*N* situations adolescents (%)	*N* situations young adults (%)	Example code^*^
Total	234	51	183	
1. Learning and applying knowledge				
2. General tasks and demands				
3. Communication				
4. Mobility	6 (2.6)	1 (2.0)	5 (2.7)	'Hearing broadcasted messages in the train' (P12)
5. Self‐care	9 (3.8)	*X*	9 (4.9)	'Talking to the podiatrist' (P07)
6. Domestic life	14 (6.0)	*X*	14 (7.7)	'Asking a question to the store employee' (P02)
7. Interpersonal interactions and relationships	86 (36.8)	28 (54.9)	58 (31.7)	
*Friends*	*38*	*16*	*22*	'Telling my opinion to a friend' (P09)
*Parents/siblings*	*19*	*6*	*13*	'Telling my parents about my day' (P11)
*Unfamiliar people*	*15*	*X*	*15*	'Calling with people I don't know' (P01)
*Intimate relationships*	*2*	*1*	*1*	'Talking to my boyfriend' (P09)
*Family*	*9*	*2*	*7*	'Communicating with grandparents' (P04)
*Other young people*	*3*	*3*	*X*	'Talking to my sister's friends' (P09)
8. Major life areas	95 (40.6)	17 (27.5)	78 (42.6)	
*School*	*50*	*17*	*33*	'Understanding the teachers explanation' (P11)
*Work*	*43*	*X*	*43*	'Participating in discussions during work meetings' (P03)
*Internship*	*2*	*X*	*2*	'Communicating on internship with colleagues' (P02)
9. Community, social and civic life	24 (10.3)	5 (9.8)	19 (10.4)	
*Community life*	*2*	*X*	*2*	'Online meeting with patient association' (P03)
*Sports*	*6*	*5*	*1*	'Understanding the instructions of the hockey coach' (P09)
*Arts and culture*	*2*	*X*	*2*	'Auditioning for a play' (P02)
*Socializing*	*13*	*X*	*13*	'Ordering drinks during a concert' (P01)
*Religion*	*1*	*X*	*1*	'Understanding the pastor in church' (P04)

Most of the situations were found in domains 7 and 8. As these domains are quite broad, it was decided to divide the situations into sub‐domains to better indicate what the situations were about. In domain 7, most situations were related to interacting with friends, parents/siblings and strangers. However, talking to strangers was only mentioned by the young adults, not by the adolescents. In domain 8, many situations related to school were discussed for both age groups. Young adults also mentioned situations related to work.

When the situations are separated into those mentioned by adolescents (*N* = 51) and those mentioned by young adults (*N* = 183), a few differences become apparent. First, domains 4, 5 and 6 are rarely mentioned by adolescents. Second, young adults mention many items in the subdomain of work (*N* = 43) that are not mentioned by adolescents. Finally, within domain 9, adolescents mention only situations related to sports, whereas young adults discuss sports as well as other activities, such as arts and culture, community life, and religion.

### 3.2 Relevant Aspects of Communicative Participation Situations

Two hundred twenty‐seven aspects that influenced the communicative participation situations were coded from the transcripts. These aspects were grouped into concepts, resulting in 37 concepts of communicative participation. Thirty‐six concepts were categorized within one of the six categories (person, topic, location, moment, mode) of communicative participation described by Ter Wal et al. (2023). An example of this coding process is provided in Table 3. The overview of concepts categorized in categories is presented in Table 4. One concept called *compensation techniques and tools* could not be divided into one of the categories. This concept is about the use of compensation techniques in conversations that facilitate participation. For example, asking for clarification from the teacher in case of misunderstanding or the use of hearing aids enabling participation. It runs through all categories and is worth mentioning separately. All categories and their meaning, and the remaining concept are described below.

**TABLE 3:** Coding process from diary to aspects, concepts and categories.

Diary situation	Situation *(Unit of analysis)*	Aspects *(Inductive codes)*	Concepts *(Inductive grouping)*	Categories (Ter Wal et al. 2023) *(Deductive categorization)*
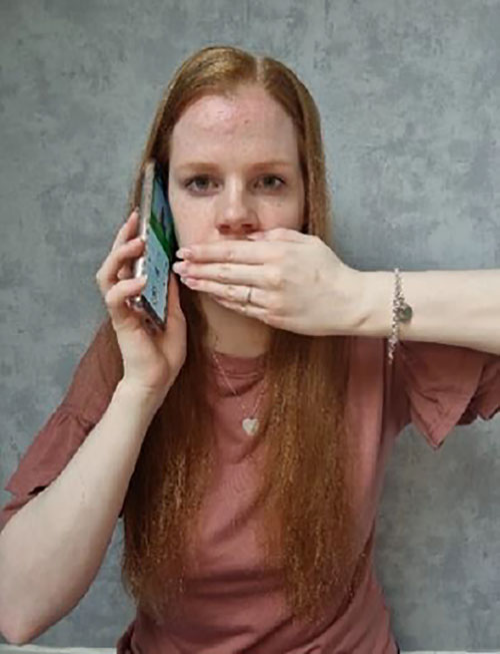	If I have to call someone, for example to the doctor or something, to the assistant, she knows I have verbal dyspraxia (*aspect 1*) but for example to another practice they don't understand me at once. Of course that makes sense because you don't know how I talk. So then it is more difficult for some people to understand me. Others do understand me better if they have had an experience like that *(aspect 2)*. But in general they find it difficult to understand me because of course I also talk and articulate differently. […] But with me, if I have someone on the phone, for example, I don't have the face to go with it. […] Does that person understand me at once? But you can't see that on a phone call like that. So no, that's why it is difficult for me *(aspect 3)*.	Calling a familiar doctor's assistant. Calling an unfamiliar doctor's assistant. Making phone calls is extra difficult, because you cannot see each other.	Health care professional Familiar person Stranger (Video)calling	Person Mode

*Note*: Photo is printed with permission of the participant.

**TABLE 4:** Identified concepts of communicative participation in adolescents and young adults.

	Person	Topic	Pace	Location	Moment	Mode
What concepts describe communicative participation in adolescents and young adults with communication disorders?	Way of speaking communication partner	Emotional conversations	Time pressure	Surroundings and acoustics	Energy	(Video)calling
	Communicate with people with communication problems	Humour		Crowded areas	Own emotions and feelings	Group conversation
	Foreign language use	Figurative language		Distance		One‐to‐one conversations
	Nonverbal communication of communication partner	Predictability of the subject		Religion		Broadcasted messages
	Familiar people	Amount/complexity of information		Work		Texting
	Stranger	Opinion/discussion		Store		Written information
	Feeling connected to a communication partner			School		Presentations
	Health care professional			Restaurant or cafe		Visual examples
	Family					Physical conversations
	Friends					
	Other young people					
	Compensation techniques and tools					

The interviews were conducted in Dutch. Quotes were translated into English, and we aimed to capture both the intended meaning of the quotes, and the language form. The following situations provide examples from participants using quotes, which means some of the quotes have errors in grammar or word choice. Those are a direct reflection of the Dutch mistakes made by the participants.

#### 3.2.1 Person

Many of the concepts that affect communicative participation have to do with the person being spoken to (in linguistics also known as interlocutor) and apply to all participants. Three forms of person can be distinguished. First, it matters to adolescents and young adults what social role they have in relation to the other person. This is described by the concepts of *familiar people, strangers, health care professionals, family, friends, and other children*. Conversation can be easier or more difficult depending on the person you are talking to. Participants often found it much easier to talk to people they knew than to people they knew less well. Even within the circle of acquaintances, it was important what social role the other person had in relation to the participant. Parents were often the unconditional know‐it‐alls and, therefore, one of the easiest people to talk to. For example, in the following two quotes:

*Pp5*: *Depends on who I'm with. Because my parents are used to it, so I don't have to pay attention to my speech*.
*Pp4*: *It does make a difference whether I am explaining to a new, unfamiliar colleague or a known colleague. That I then have to give explanations on his first working day. It does get better the more often I speak to him*.

Second, it matters in what way the other person communicates. Some participants described that despite one person having a similar social role, communication could be experienced differently because of the way the other person communicates. Is he or she able/willing to adapt his communication? Does the communication partner ask again when the message is unclear? Is the partner also having a communication problem? This is described by the concept of *Way of speaking communication partner, Communicate with people with communication problems, Foreign language use, Nonverbal communication of communication partner*.

*EA*: *And is that different whether you were talking to him or you were talking to your friends?*
*Pp8*: *Yes. I felt like if he just understood me right away, understands. And if I… Yeah. If he didn't understand me so well, he would try to think it over and then calmly…. To ask in a calm way what do you actually mean by that?*
*EA*: *Yes*
*Pp8*: *And my friends are like: why do you not understand me already? So it really depends on which friend you're talking to*.

Last, participants indicate that it is helpful when they are in conversation with someone with whom they feel a connection, described by the concept of *feeling connected to communication partner*. This was mentioned by only participants with DLD (Pp1, Pp6 and Pp7). Participants mentioned that when they, for example, knew that their communication partner (both familiar people and strangers) had something in common (e.g., being creative or spiritual) or had an enthusiastic appearance, they would feel more connected to this person, which made it easier to start a conversation.

*Pp6*: *I attended an interview at what might be my new job. Not sure. I went for an interview with two bosses, boss lady and boss, which went really well. They really clicked and talked right away and yes. They also knew a bit about the creative side. That went creative side as well. And I really enjoyed talking to them*.
*EA*: *Why is that?*
*Pp6*: *I think just the energy. I always have it when someone is a bit spiritual or something like that. […]But if someone is open and just talks well and also wants to grant you things and let you talk. Those are people I just click with more. Just… then we'll chat a bit and that's really fine with me. That's when I feel comfortable.[…] There are people who are unpredictable. Then I don't know what to say and then I have to wait a while and then I think of what how are you going to react that?*

Another example is provided from the interview with Pp7, in which the participant described that she liked it better to listen to an enthusiastic teacher than a boring teacher:

*EA*: *And then when someone is so enthusiastic, I can imagine that's more fun to listen to anyway, but does it also help you in better understanding what is being said?*
*Pp7*: *Yes sometimes it does. It really sometimes really has to do with the person appearance. For me. Then if there's such an emotionless person in front of me and then maybe they explain well or not at all, I'm like yeah, never mind. And if the person is more enthusiastic and really wants to help and so on, I'd rather ask again. Because then I know that they are prepared to help you in that situation. And then you really think like yes, then I can ask you a few… a few more questions*.

#### 3.2.2 Topic

The topic that is being discussed is of influence on the degree of self‐perceived communicative participation, and this category was found in all participants. Two forms of the topic were found. First, this category includes the topic itself, described by the concepts of *Emotional conversations, Humour, Figurative language, Opinion* and *Discussion*.

*Pp2*: When *I start talking about emotional topics like that, because I can say a lot of things spontaneously, but as soon as it comes to certain topics, then. Yes, I just get some kind of blockage, then I just can't come up with it. I don't know what will. I don't know what. I know what I want to say, but then again I don't know what I want to say. And then I get frustrated because I just really can't come up with those words then. Sometimes I have the words really stuck in my head and then I just can't get them out*.

Second, participants mentioned more generic factors influencing the complexity of the topic, described by the concepts *Predictability of the subject* and *Amount/complexity of information*.

The following quote reflects a comparison of having a spontaneous conversation with colleagues at work versus a substantive conversation about work, which shows how the complexity of the topic is of influence on the degree of self‐perceived communicative participation.

*Pp4*: *The spontaneous conversation. That goes better. But a conversation where it's really about work content. That always is a bit more difficult. Them I'm more concerned with the content of the topic and uh. I'm thinking a lot more. And that does not help either*.

#### 3.2.3 Pace

The pace in which the conversation takes place is also of influence on the degree of self‐perceived communicative participation and is described by the concept of *time pressure*. Time pressure was a frequently mentioned factor that negatively affects the self‐perceived communicative participation of the participants. This was mentioned by nine participants and across all different communication problems. Time pressure was mentioned negatively, as the participant was not given enough time to speak, read or write, but also when the communication partner spoke too fast, giving the participant too little time to understand the message.

*Pp1*: *Well, I look at whether the store employee is busy and needs to finish his work quickly or whether he is just standing at the cash register and working and has nothing to do. Then I speak to him before I speak to someone who is busy […]. Because when I talk to someone, he has to have some time to understand me, how I talk and what my question is. And if I don't have time, I feel that the person can't really understand me*.
*Pp7*: *Yes in the beginning, driving lessons were really hard because then I had to do everything at once myself. […] But the driving instructor there is very good and he also explains things just very well. And very… Yes… He's very*.
*Mother7*: *He's really very calm and that works very well for you*.
*Pp7*: *Yes*.

#### 3.2.4 Location

Participants reported difficulty in their communicative participation that can be clustered within the category location. Almost all participants mentioned location as an influencing factor, and the subgroups of participants with hearing loss and DLD mentioned it very often. The location being of influence was mentioned in two ways. First, participants mentioned specific locations from everyday life, described by the concepts of *religion* (the church)*, work, store, school, and restaurant or cafe*.

*Pp3*: *I belong to a student society and we have set groups with which you eat together on Tuesday nights for a year […]. And that night we went out for dinner, because it was the last time. And we were in a restaurant, Greek restaurant where the music was pretty loud. Well restaurants are spacious now because there have to be a lot of people in there as well […]. Um, so that made it difficult for me to understand. And restaurants are often a problem, even when there is no music, it is also very noisy. Then I really have to do my best, so to speak*.

On the other hand, they also mention certain factors that are not linked to a specific location, but can be seen as an environmental factor affecting the location that influences the communicative participation. This is described by the concepts of *Surroundings and acoustics, Crowded areas*, and *distance*.

*Pp5*: *In crowded places, I can never understand others. Because I have a little bit of hearing loss, not a lot, but I notice it especially there. This makes me less inclined to seek out or initiate interaction with people. But it is also more difficult for other people to understand me if there is a lot of background noise*.

#### 3.2.5 Moment

For the participants, experienced problems depended on what moment they had to have a conversation. Two concepts describe the influence of the moment on the communication. First, the concept of *energy* is about being tired or having less energy, making it more difficult to participate in a conversation. It makes it difficult to follow a conversation or pay attention to speaking slowly or loudly enough.

*Pp7*: *When I am a bit tired, it's harder to understand. […] We had just returned from a weekend with my father's side of the family. Were a weekend away. And that's just exhausting for me. And then I had driving lessons the next day and everything didn't go well. And when everything doesn't go well in my head, I just want to do better and improve and so then I create a kind of confusion so an errors in my head and then I just do it wrong. […] And my driving instructor saw it in me and said hey what's up, are you tired? Yes I am tired. My head doesn't want to be there today*.

Second, moment also refers to the concept of *own emotions and feelings*. This concept is not about talking about an emotional or sensitive topic, but about talking in general when you feel emotional, nervous, insecure, and so forth.

*Pp6*: *Well I am a morning person but I am also sometimes I am not a morning talker. […] I had also sometimes had a situation where I would come out of my bedroom and my mother would say, oh you're getting up would you like to make me a cup of coffee? … Ehm so ehm then I'm like yes is good but then we also went for a chat. Then I'm like oh I don't feel like that. It's morning, I just woke up*.
*EA*: *And so is talking harder for you when you've just got up?*
*Pp6*: *Um it depends, if I've just opened my eyes and I've just got up it's harder because then I really have to search for sentences. Then I need more time. If I wake up normally and I'm already going out the door, I've already had breakfast, then it's totally fine*.

#### 3.2.6 Mode

Participants mentioned that for them, the mode in which the conversation takes place, influences their degree of communicative participation. A few subgroups can be formed of concepts that have a connection with each other.

Participants mentioned difficulties in one‐to‐one conversations and in a group conversation. This was found across all types of communication disorders. Although both were often mentioned as difficult, participants expressed that group conversations were more difficult to participate in than one‐to‐one conversations. However, for some participants, even one‐to‐one conversations were difficult:

*Pp9*: Because *when there are two of you then the other wants so much…. When sleeping over… she wants so much… What do you call that? Then they want to do so much in the evening. Does your head get full very quickly. So you then get angry at that. […] But if there are more of you, so three. Again, that can also… Unlucky. Because. Then you might also have fun, only then you are usually ignored. Then it looks like you're being ignored*.

Participants mentioned that many conversations took place digitally via (video)calling. They often made the comparison between these digital conversations versus physical conversations, and mentioned experiencing digital conversations as more difficult, as there is less to no nonverbal communication.

*Pp8*: *I do notice generally with calling that sometimes I stutter a bit more often or. Then um. Then just, yes, 1‐1 or with a group of people. […] I think also with facetiming. That might also be a bit easier. But I never really have much trouble with it. But it's still a bit different from actual calling*.

Communication that takes place through written information was especially difficult for participants with DLD and is described by the concepts of *texting* and *written information*. For example, in this quote where we talked about texting by phone:

*Pp7*: *Yes but I try to type most of the time, you know. That goes pretty well too. But it's mostly short sentences. And then my friends send me all these long texts and then I really think like. Yeah, what now? Can you make it a bit shorter?*

Another concept that was only mentioned by participants with language difficulties was the concept of *visual examples*. This refers to participants seeing a visual example when hearing or reading a message, which facilitates the comprehension of the message. A concept that was mentioned by only one participant (pp11, hearing problems) was the concept of *broadcasted messages*. Due to hearing problems, the participant did not hear the train conductor and did not know if she had arrived at her final destination.

*Pp11*: *Yes, because we were in the train and it was very busy. And I wanted to go to [name destination]. And when the train stopped, people talked very loudly and I did not hear what the conductor said. So we went to the conductor and asked what did you say, it was very loud over there. And I asked him if we were in [name destination] already and he said yes. So then we got out of the train*.

#### 3.2.7 Compensation Techniques and Tools

One concept called *compensation techniques and tools* could not be divided into one concept or theme that influences communicative participation. This concept did not describe one specific element of communicative participation, but was about strategies or solutions people with the communication disorders use to compensate for their problems, which enables them to participate. The strategies mentioned varied, such as supporting signs, gestures, asking for repetition, and using hearing aids. Participants often applied these strategies so naturally that they did not even think about them. This concept affects every category, and using them facilitates the participation greatly.

*Pp7*: *Texting goes pretty well. Only sometimes when I can't figure out a word, I just press the speaker for the wordmaker (dictation function). And then, for example, I say the word and he writes it for me at once and then I don't have to type it in. […] And then I do like: I want to say this and this then it types out my whole thingy. So that's… That's really helpful*.

### 3.3 Personal Character

In addition to the concepts and the categorization of the concepts within the elements, there is one other finding worth mentioning. Some participants had difficulties reflecting upon their communicative participation in relation to their communication disorder. It was difficult to distinguish whether a participation problem was related to their communication problem, or whether it was part of their character.

*Pp5*: *I don't really know. Also because I… um… am not always very social myself, for me it is sometimes very much the question whether I don't dare because of my character or I don't dare because I am afraid that people won't understand me*.
*Mother Pp12*: *Because you don't know better than to have DLD. So you have to actually come across something of saying ah, that's where it went wrong or doesn't work. That for you was thinking of…. Where does DLD bother me*.
*Pp2*: *But I can also remember sometimes finding it difficult to connect with other children, but what child doesn't have that, you know*.

## 4 Discussion

The aim of this study was to identify relevant, self‐experienced situations in which adolescents and young adults with speech, language, hearing and voice disorders have difficulty participating because of communication problems, as well as situations in which participants have no difficulties. In addition, we aimed to identify relevant aspects of these situations that influence communicative participation. The 12 participants mentioned a total of 234 situations. Out of these situations, 37 concepts that influence communicative participation were elicited, related to six categories: *person, topic, pace, location, moment*, and *mode*.

### 4.1 Participation Situations

The communicative participation situations mentioned by participants can be categorized in 6 out of 9 domains of the ICF Activities and Participation chapter (domains 4, 5, 6, 7, 8 and 9). In a systematic literature review (Alons et al. 2024), which searched for items measuring communicative participation from existing PROMs and parent reports, items were categorized into the ICF domains as well. When comparing the results of the covered domains, some differences become apparent. In the literature review, it was found that existing items covered only 5 ICF domains (domains 5, 6, 7, 8 and 9), and most items were related to domain 7: interpersonal interactions and relationships. The found items were generally not developed from the perspective of children and adolescents themselves. In this study, we focused on the adolescents’ and young adults’ perspectives and found different results. First, adolescents and young adults mentioned situations related to domain 4, mobility, a domain that was not found in the literature review. These situations refer to participation situations in which communication is needed when travelling by public transport. Second, more situations were found referring to domain 8, which is about school and work. This domain was covered in the literature review, but only 13.8% of the items were about communicative participation in school, compared to 40.6% in this study. Including the perspectives of adolescents and young adults brings focus to additional areas that are prioritized by youth, thereby expanding our understanding of their experiences.

In this study, we aimed to recruit at least 12 participants, representing both adolescents and young adults. Adolescents mention none or only a few situations of ICF domains: mobility (travelling by public transport), self‐care (communicating with a doctor, making appointments at the dentist), and domestic life (asking a shop assistant for something). It could be that these situations are not yet frequent in adolescents’ lives, or that they always participate in these kinds of situations with an adult (e.g., parent) present. As a result, these situations may not be considered very important from adolescents’ perspectives. Within ICF domain 9 Leisure, adolescents only mention examples of situations involving sports. Young adults, however, also mention situations where they engage in social contacts in public places, such as cafes, restaurants, bars or discotheques. Although the adolescent group consisted of only three participants, and these results should be interpreted with this in mind, it can be assumed that an individual's social context grows as they grow up, leading to a larger set of relevant situations.

Comparing this finding with the descriptions of life stages according to the LCHD framework, this growth is also evident (Wood et al. 2017; Arnett 2000). Becoming a young adult means moving out on your own for the first time, starting a relationship and setting out on a clear career path (Wood et al. 2017). Young adults start developing the ability to become self‐sufficient, assume more adult roles and responsibilities and obtain a level of education and training that sets the foundation for work during the adult years (Arnett 2000). Within the ICF domains 4 Mobility, 5 Self‐Care, and 6 Domestic Life, a young adult may report on more participation situations than an adolescent.

### 4.2 Concepts Describing Communicative Participation

For the second research question, the situations mentioned by the participants were further explored. This led to concepts describing communicative participation. Our approach was based on the concept elicitation study by Ter Wal et al. (2023), who conducted a similar study with adults. There was one important difference in method: Ter Wal et al. (2023) asked participants about situations in which they had difficulty participating because of their communication problems. In this study we asked about difficult situations, but also situations in which they had *no* difficulties. This, combined with the fact that we focused on a different target population, led to the identification of new concepts that were not found in the concept elicitation study in adults (Ter Wal et al. 2023).

We found a concept called compensation techniques and tools. This concept refers to the use of strategies, techniques and tools that participants mentioned as helpful in their communicative participation. Sometimes, participants no longer experienced issues with participation because they had become so used to the application of compensation techniques or using some kind of tool. They do not know better than to have to use these compensation techniques and often do not experience it as difficult or problematic. By asking also about positive experiences with communicative participation, and, for example, by asking about situations in which a particular technique is used, we gained more information about important concepts around communicative participation.

Furthermore, we found an additional concept within the element person: feeling connected to the communication partner. In the interviews, participants experienced better communicative participation when they felt a connection to the communication partner. This concerned both acquaintances and strangers. The connection was based on common interests (e.g., spirituality or creativity) and made it easier for them to talk about a topic. They were more likely to feel they could arrive at a shared meaning in the conversation. In literature, this topic is described as having common ground: a set of shared knowledge, beliefs and assumptions that exists between two speakers (Clark 1996). Feeling common ground with a conversational partner provides a relief of the communication burden (Doedens and Meteyard 2020).

Compared to Ter Wal et al. (2023), some differences in concepts were identified, reflecting the participants' different life stages. For instance, in our study with adolescents and young adults, we identified the concept of *school* within the category of *Location* and *other children* within the category of *Person*. In contrast, Ter Wal et al. (2023) found concepts such as *email*, *personal mail*, and *meetings* in the category *Mode*, as well as *administration* in the category *Topic*. These differences align with the participants’ life stages and underscore the importance of developing separate measurement instruments that account for such variations, as also recommended by Darling‐White (2017).

This study aimed to explore situations that might be relevant to include in an outcome measure for communicative participation, aligning in some ways with the work of Markhamet al. (2009). Markham and colleagues conducted a qualitative study on the quality of life experiences of children and young people (6–18 years old) with speech language and communication needs, ultimately aiming to create a quality of life measure. Both studies share similarities in their goals—developing outcome measures—and in their inclusion of participants with diverse ages and communication challenges. However, the central construct of interest differs: while Markham et al. (2009) focused on quality of life, our study centred on communicative participation. There is some overlap between our findings and Markham's identified themes, such as *emotions*, *relationships*, and *school*. Markham's corresponding codes (e.g., *confidence*, *family*, *friendships*, *teachers*, and *noise*) also align with concepts we identified as influencing communicative participation. However, Markham et al. identified additional codes, such as *achievement*, *anxiety*, *frustration*, *bullying*, and *lonely*, which we did not observe. This discrepancy may be due to the broader scope of quality of life as a construct. Some of Markham's codes reflect the consequences of (un)successful participation (e.g., bullying or frustration) rather than describing participation itself. This distinction is critical to consider in both clinical practice and the selection of outcome measures for scientific research.

### 4.3 Clinical Implications

Previously conducted research that focuses on obtaining perspectives of adolescents and/or young adults with a variety of communication problems has shown that successful communication is not only about the communication disorder, but also about personal factors and environmental factors that need to be taken into account (Zaidman‐Zait and Dotan 2017; Ekström et al. 2023; Cobb et al. 2019; Connaghan et al. 2022; Kelly et al. 2018). There is a need for a holistic approach in the field of SLT, in which it is essential to understand the complex interaction between communication abilities, personal factors and environmental factors (Ekström et al. 2023; Cobb et al. 2019; Connaghan et al. 2022). We believe that this study forms a first step towards this holistic, person‐centred approach. Where the previous research focuses on obtaining themes to obtain deep understanding, we focused on a more broader approach by obtaining key concepts that influence communicative participation and explicitly with the goal to create items for a PROM.

This study was carried out to obtain adolescents' and young adults' perspectives on communicative participation to inform the development of a PROM. As the results show, capturing a complex construct such as communicative participation is challenging, as communicative participation involves many factors at different levels that may be experienced differently by individuals. A PROM will be a valuable addition to the clinical practice of speech and language therapy, and can enhance the conversation between the client and the SLT about what factors apply to the life of the client and to what degree (Gibbons et al. 2021). The overview of all concepts can provide guidance for the SLT to start the conversation about this topic as a first step towards person‐centred, participation‐focused therapy.

Although the group of adolescents was relatively small compared to the group of young adults, we found differences between the identified situations between the two groups. Some situations mentioned by the young adults were not mentioned by the adolescents. It is therefore worth considering developing two separate instruments adapted to the context of adolescents or young adults. However, this decision cannot be based on information from only three adolescents.

### 4.4 Study Limitations

Although we believe this study is informative and a good step towards the development of a tool that measures communicative participation, there are some limitations worth mentioning. First, despite many attempts to recruit a representative sample with at least two participants per type of communication disorder (speech, language, hearing or voice), we did not succeed in including participants with voice disorders. In addition, seven participants had the diagnosis DLD. The results presented in this study therefore contain an over‐representation of people with DLD and under‐representation of people with voice disorders. The perspective of adolescents and young adults with voice disorders was not included in the results of this study, and thereby in the first phase of the PROM development, which is a clear limitation of the study. However, earlier research on adults shows that adults with different communication problems describe similar communicative participation problems (Baylor et al. 2011). In their paper, Baylor and colleagues present an example of participation in a loud environment, in which people with speech and voice disorders experience problems in not being loud enough, and people with other communication problems report on following the conversation and being distracted by all the noise. In terms of the first research question, it is therefore assumable that we would not have found different participation situations. In terms of the second research question, it is possible that we missed out on some concepts that describe the difficulties from the perspective of individuals with voice problems. More attention to this subject is required. In terms of further developing the PROM, extra attention will be paid to testing the comprehensiveness of the PROM among individuals with voice disorders.

Second, it was difficult to recruit the group of adolescents, which caused only three adolescents to participate. We were unable to recruit participants aged between 15 and 17. Some of the potential participants declined to participate, stating that they did not want to be associated with their disorder and simply wished to be treated equally to their peers. Unfortunately, this means that we missed out on a relatively large group, and their perspectives were not fully captured. This limitation is acknowledged in this study.

Third, in this study we included a wide group of individuals with great variation in type of communication disorder, keeping in mind that results would eventually inform a generic PROM that is applicable to a large group of people, also with variation in nature and severity of communication problems. As such, the current study adds to the literature. However, one could also see this variation as a lack of depth within certain subgroups of our population.

### 4.5 Conclusions and Future Directions

The results provided us with an overview of 234 self‐experience participation situations that require communication, from which we identified 37 concepts describing communicative participation. This led to more insight into the perspective of adolescents and young adults with communication problems on communicative participation. The results will be used to develop a PROM (in the form of an item bank) to measure communicative participation in children, adolescents and young adults.

This study shows that adolescents and young adults find different situations important than described in a literature review of existing items on communicative participation (Alons et al. 2024). While it is acknowledged that the target population for whom a measurement instrument is being developed should be involved, the comparison between this study and the literature review highlights the necessity of involving children, adolescents, and young adults themselves (Terwee et al. 2018; Patrick et al. 2011). Only by doing so can we ensure that the measuring instrument accurately measures what is important to the target population. The identified communicative participation situations form the basis for developing an item bank for measuring communicative participation in adolescents and young adults with communication disorders.

The mentioned situations will be combined with items found in literature (Alons et al. 2024), resulting in a first draft of an item bank. In upcoming studies, the items will extensively be tested on quality aspects comprehensiveness, comprehensibility and relevance in adolescents and young adults separately, following the PROM development steps outlined by the COnsensus‐based Standards for the selection of health Measurement INstruments (COSMIN) initiative (Terwee et al. 2018).

Another important future direction is the inclusion of children under the age of 12. Research on this target population is ongoing and will be reported on in a separate paper.

## Conflicts of Interest

The authors declare no conflicts of interest.

## Data Availability

The data that support the findings of this study are available from the corresponding author upon reasonable request.

## Appendix 1

### Explicit Questions Asked in the Diary

**Table:**

What did you do today? What activities did you do in the
○ Morning
○ Afternoon
○ Evening
In what situations were you able to participate well? In what situations was participation difficult?

### Interview Guide

**Table:**

**Warm‐up question** How did you experience keeping the diary? ○ Were there any difficulties in keeping the diary? **Topic 1: Diary content** Can you describe the situation you were in? What makes this situation easy/difficult? What could help you in this situation? How often does this situation occur?
**Additional questions**
We have now talked about the situations from your diary. Are there other situations that did not occur in your diary, in which you find it difficult to participate because of your communication problems? If you compare yourself to someone your age without communication problems, are there things you don't do because of your communication?
